# Patients with transplantation have reduced mortality in bacteraemia: Analysis of data from a randomised trial

**DOI:** 10.1016/j.jinf.2022.05.014

**Published:** 2022-07

**Authors:** Fergus Hamilton, Rebecca Evans, Peter Ghazal, Alasdair MacGowan

**Affiliations:** a Infection Sciences, Pathology, North Bristol NHS Trust, Bristol, United Kingdom; b Population Health Sciences, University of Bristol, Bristol, United Kingdom; c Bristol Trials Centre, Bristol Medical School, University of Bristol, Bristol, United Kingdom; d Project Sepsis, Cardiff University, Cardiff, United Kingdom

**Keywords:** Bloodstream infection, Immunosuppression, Mortality, Transplant

## Abstract

**Objectives:**

Infection remains a major complication of organ transplantation. Paradoxically, epidemiological studies suggest better survival from serious infection. We analysed the relationship between organ transplantation and short -term mortality of patients with bloodstream infection.

**Methods:**

Data on transplantation status was extracted from a large prospective, multi-centre clinical trial in bloodstream infection. Logistic regression for 28-day mortality was performed on the whole cohort and a propensity-matched cohort (3:1). Infective pathogen, focus of infection, and clinical variables were included in the model. Mediation analysis was performed on clinical variables to explore causation.

**Results:**

4,178 participants were included in the full cohort, with 868 in the matched cohort, of which 217 received an organ transplant. Haematopoietic stem cell transplants (HSCT) were the most common transplant (*n* = 99), followed by kidney (*n* = 70). The most common pathogens were staphylococci and *Enterobacterales*. Transplantation status was associated with a reduced mortality in both the whole (Odds Ratio, OR 0.53; 95% CI 0.28, 0.77) and matched (OR 0.55, 95% CI 0.34, 0.90) cohort, while steroid use was robustly associated with increased mortality OR 4.4 (95% CI 3.12, 6.20) in the whole cohort and OR 5.24 (95% CI 2.79, 9.84) in the matched cohort. There was no interaction between steroid use and transplant status, so transplant patients on steroids generally had increased mortality relative to those without either.

**Conclusions:**

Organ transplantation is associated with a near halving of short term mortality in bloodstream infection, including a cohort matched for comorbidities, infective pathogen and focus. Steroid usage is associated with increased mortality regardless of transplant status. Understanding the mechanism and causation of this mortality benefit should be a focus of future research.

## Introduction

Although organ transplantation has been dramatically successful in improving outcomes in multiple settings, infection remains a major complication. In particular, infection in patients with haematopoietic stem cell transplant (HSCT) remains a major determinant of mortality, especially in the peri‑engraftment period.^1^

However, the data from solid organ transplantation and infection outcomes remains less clear. Although many studies have shown that the rates of infection are higher in patients with transplant,^2^^,^^3^ the data on outcomes in individual infection episodes is equivocal. A recent multi-centre study of transplant patients with pneumonia had a surprisingly low in-hospital mortality rate of 1.9%,^4^ while two case-control studies have suggested that the mortality of patients with a transplant and with severe sepsis or bloodstream infection is significantly lower than age matched controls.^5^^,^^6^ In contrast a population based study from Denmark found a markedly increased rate of pneumonia in renal transplant patients, but similar mortality for a given pneumonia episode.^3^

In this study, we aimed to estimate the absolute and relative risk of short term mortality in patients with transplant using data from a large, prospectively collected, cohort of patients with bloodstream infection.

## Methods

### Objectives

Our primary objective was to estimate the effect of transplantation status on outcome in patients with a bloodstream infection, in a large, representative cohort of UK bloodstream infection in England and Wales.

Our secondary objective was to estimate whether any benefit was mediated by changes in clinical features.

### Data source

All participants included in this study were part of the RAPIDO trial, an NIHR funded randomised controlled trial on the impact of rapid identification of bloodstream infection organisms by matrix-assisted-laser-deionisation time of flight (MALDI-TOF) analysis across seven NHS laboratories in England and Wales. This paper was recently published and found no evidence of a mortality benefit.^7^ Ethical approval for the RAPIDO study was granted by the National Research Ethics Committee South West (12/SW/003; First MREC approval date 20/03/2012).

Full details of the inclusion criteria are published with the original trial.^7^ Briefly, all adult patients in the seven included NHS sites who had a blood sample culture positive for bacteria or fungi between July 2012 and August 2014 were potentially eligible for inclusion. The exclusion criteria of the trial were: patient not receiving NHS care; not admitted to hospital when, or shortly after, the blood sample was taken; under 18 years old; in custody; on an end-of-life care pathway when the sample was taken; or, exceptionally, judged unsuitable by the attending physician.

Clinical information (including transplantation status) was recorded by the trial nurse on entry to the study, and based on the medical history and clinical notes. Full details on clinical variable definition are recorded in the original trial.^7^ Hospital acquired infection was defined as any positive blood culture from a sample taken >= 2 days after admission. Corticosteroid use was defined as receiving systemic corticosteroids on day 0,1, or 2.

### Analysis population

All consented patients in the RAPIDO trial were included in this analysis, except where information on transplantation status or corticosteroid use was not available.

### Analysis methodology

For our primary analysis, we used multivariable logistic regression with an exposure variable of transplantation status and an outcome of 28-day mortality from blood culture collection. We used multiple approaches to account for potential confounding.

Firstly, we performed 3:1 propensity score matching (within imputed cohorts) using a nearest neighbour approach in the *MatchThem* package in R, to generate a cohort matched for age, Charlson's Comorbidity Score, gender, infective pathogen, NHS site and receipt of recent chemotherapy (within the last month).

Subsequently, we fitted a multivariable logistic regression (using the R function *glm*) adjusting for the same comorbidities and hospital acquisition of infection. We did not include clinical features and hospital acquisition of infection in the propensity matching as they occur downstream of transplant status and might have biased causal estimates.

For robustness we also performed the analysis as a univariate model, and replicated all analyses on the whole cohort before propensity score matching, to assess the impact of the matching process.

To explore whether the type of transplantation was relevant, for transplants with enough cases (*n* > 20), we performed an additional analysis with transplantation type as a factor variable.

For the secondary objective, we used logistic regression models to assess whether clinical variables on presentation differed between patients with and without transplantation status and whether these changes mediated any of the effect of transplantation status. We performed multivariable mediation analysis using the R package *mediate* and estimated the direct and indirect causal effects of these variables.

As a subsequent analyses, we evaluated the potential effect of both transplantation status and corticosteroid usage. Firstly, we fitted a model with an interaction between steroid usage and organ transplantation, to evaluate whether steroid usage had a differing effect in organ transplant patients. Subsequently, we estimated the relative effect of each by comparing three groups (steroid and transplant; steroid and no transplant; no steroid and transplant) against the reference (no steroid and no transplant).

In all analysis, we imputed missing data using multiple imputation (5 imputations) using the *mice* package in R, with chained equations.

### Role of the funding source

The funder of this study (the NIHR) had no role in design, analysis, or conception of the study, writing of the study, or the decision to submit for publication.

## Results

### Demographics and matching

4468 participants were included in the RAPIDO trial, of which 290 had missing data related to transplantation status or corticosteroid use, leaving 4178 participants in this analysis. Of these participants, 217 (5.2%) were recorded as having a transplant. The commonest transplant was a haematopoietic stem cell or bone marrow transplant (*n* = 99), followed by kidney (*n* = 70), liver (*n* = 26), heart (*n* = 4), lung (*n* = 4), kidney and pancreas (*n* = 3), kidney and lung (*n* = 1), and pancreas (*n* = 1). Nine patients did not have the type of transplant recorded. Table 1 describes the demographics and clinical status of the cohort, by transplantation status, while Table 2 describes the microbiological data. Missing data was most common in the clinical variables.

**Table 1:** Demographics and clinical status of the included cohort.

	No transplant *n* = 3961	Transplant *n* = 217	p-value^1^
Gender			0.024
Female	1788/3961 (45.1%)	81/217 (37.3%)	
Male	2173/3961 (54.9%)	136/217 (62.7%)	
Age	70 (56, 81)	57 (46, 66)	<0.001
Cardiac arrest up to day −7	65/3960 (1.6%)	1/216 (0.5%)	0.3
Antineoplastic chemotherapy in last month	397/3960 (10.0%)	71/216 (32.9%)	<0.001
Surgery requiring overnight stay up to day −7	329/3960 (8.3%)	22/217 (10.1%)	0.3
Neutrophil count on day 0	9.4 (5.7, 13.8)	4.2 (0.2, 9.6)	<0.001
Ventilated on day 0	340/3954 (8.6%)	16/216 (7.4%)	0.5
Temperature on day 0	38.1 (37.2, 38.7)	38.1 (37.5, 38.6)	0.4
Systolic Blood pressure on day 0	120 (105, 139)	125 (105, 140)	0.2
On IV fluids on day 0	1685/3891 (43.3%)	80/212 (38.1%)	0.12
On vasopressor drugs at day 0	322/3960 (8.1%)	16/216 (7.4%)	0.7
On corticosteroids on day 0, 1 or 2	195/3961 (4.9%)	24/217 (11.0%)	<0.001
Hospital acquired	1419/3938 (36.0%)	109/213 (51.0%)	<0.001
Charlson Comorbidity Index	3.0 (2.0, 4.0)	3.0 (2.0, 4.0)	0.030
Encephalopathy			<0.001
Grade I	165 (4.2%)	2 (0.9%)	
Grade II	242 (6.1%)	4 (1.8%)	
Grade III	405 (10%)	9 (4.1%)	
Grade IV	87 (2.2%)	1 (0.5%)	
None	3062 (77.0%)	201 (93.0%)	<0.001
Time to appropriate therapy (hours)	5.0 (1.0, 31.0)	4.0 (0.0, 26.0)	0.086
Dead at 28 days	915 (23.1%)	30 (14.0%)	0.001
1 n (%); Median (IQR)			

Missing data for neutrophil count (178; 169 no transplant, 9 transplant).

Missing data for temperature (122; 114 no transplant, 8 transplant).

Missing data for systolic blood pressure (298, 277 transplant, 19 no transplant).

Missing data for systolic Charlson Comorbidity Index (840, 807 transplant, 33 no transplant).

1 Pearson's Chi-squared test; Wilcoxon rank sum test; Fisher's exact test.

**Table 2:** Microbiological source of bloodstream infection and focus of infection.

	No transplant*n* = 3961	Transplant*n* = 217	p-value^1^
**Type of organism:**			<0.001
Coagulase negative Staphylococci	1099 (28%)	45 (21%)	
Anaerobes	30 (0.8%)	0 (0.0%)	
Candida spp	53 (1.3%)	3 (1.4%)	
Enterobacterales	1190 (30%)	74 (34%)	
Enterococci	105 (2.7%)	16 (7.4%)	
Other	344 (8.7%)	16 (7.4%)	
Polymicrobial	260 (6.6%)	18 (8.3%)	
Pseudomonas spp	109 (2.8%)	14 (6.5%)	
Staphylococcus aureus	347 (8.8%)	14 (6.5%)	
Streptococci	424 (11%)	17 (7.8%)	
**Focus of infection:**			<0.001
Bone and joint	76 (1.9%)	1 (0.9%)	
Cardiovascular system	84 (2.1%)	3 (1.3%)	
Central nervous system	24 (0.6%)	2 (0.9%)	
Eye, ear, nose, throat or mouth	17 (0.4%)	1 (0.4%)	
Gastrointestinal system	370 (9.4%)	20 (9.4%)	
Line infection - central venous line	238 (5.9%)	41 (18.9%)	
Line infection - peripheral venous line	20 (0.5%)	1 (0.4%)	
Lower respiratory tract	314 (7.8%)	15 (6.7%)	
N/A - contaminant	1159 (29%)	30 (13%)	
Reproductive tract	14 (0.3%)	0 (0.0%)	
Site uncertain	788 (19.9%)	61 (28.1%)	
Skin and soft tissue	152 (3.8%)	3 (1.3%)	
Surgical site infection	59 (1.5%)	2 (0.9%)	
Systemic Infection	16 (0.4%)	0 (0.0%)	
Urinary tract infection	664(7%)	37 (17%)	

1 Pearson's Chi-squared test.

Patients with transplantation were more likely to be male (63% vs 55%) and younger with a median age of 57 (IQR 46, 66) compared to 70 (IQR 56, 81). There were differences in the presence of comorbidities and clinical picture. Notably, patients with transplantation were more likely to have had recent antineoplastic chemotherapy (33% vs 10%) and have acquired their bloodstream infection in hospital (51% vs 36%). The infecting pathogen varied between the two groups, with Pseudomonas spp and Enterococci being more common in patients with transplant (6.5% vs 2.8% for Pseudomonas, and 7.4%vs 2.7% for Enterococci), while coagulase-negative staphylococci were less common (21% vs 28%). Importantly, neutrophil count on day 0 differed between the groups with a median of 4.2 (IQR 0.2, 9.6) in patients with transplant, and 9.4 (IQR 5.7, 13.8) in patients without. The focus of infection differed between the two groups, with a larger number of patients with unknown source of bloodstream infection in those with transplantation (29% vs 20%), a higher rate of central line infections (19% vs 6%), and a lower number of likely contaminants (13% vs 29%).

Absolute 28-day mortality for each type of transplant, and for those without transplant, is shown in Table 3. For the two commonest types of transplant, HSCT and kidney, absolute mortality was considerably lower than in patients with no transplant (13.1% in HSCT, 12.9% in kidney vs 23.1% without transplant).

**Table 3:** Absolute 28-day mortality by type of transplant.

Type of transplant	n	28-day mortality (n,%)
No transplant	3961	915 (23.1%)
Haematopoietic stem cell	99	13 (13.1%)
Kidney	70	9 (12.9%)
Liver	26	2 (7.7%)
Not recorded	5	0 (0.0%)
Heart	4	3 (75.0%)
Lung	4	4 (75.0%)
Other	9	0 (0.0%)
Kidney and pancreas	3	0 (0.0%)
Kidney and lung	1	0 (0.0%)
Pancreas	1	0 (0.0%)

The cohort was then multiply imputed and matched, in order to minimise the baseline differences in age and comorbidity. Table 4 describes the demographics of the matched cohort, while Table S1 shows the matching on microbiological source and focus. Figure S1 describes the change in standardised mean difference (SMD) with the matching process for each variable. Matching was generally successful, with a mean standardised mean difference (SMD) of 0.076 across all variables). Importantly, cohorts were matched well on infective organism and focus of infection.

**Table 4:** Post imputation and propensity score matching demographics (single imputed dataset shown).

	No transplant *n* = 651	Transplant, *n* = 217	p-value^2^
Gender			0.8
Female	250 (38%)	81 (37%)	
Male	401 (62%)	136 (63%)	
Age	55 (39, 68)	57 (46, 66)	0.8
Cardiac arrest up to day −7	23 (3.5%)	1 (0.5%)	0.017
Chemotherapy in last month	189 (29%)	71 (33%)	0.3
Surgery requiring overnight stay up to day −7	58 (8.9%)	22 (10%)	0.6
Neutrophil count on day 0	8 (4, 13)	4 (0, 9)	<0.001
Ventilated on day 0	78 (12%)	16 (7.4%)	0.059
Temperature on day 0	38.20 (37.50, 38.80)	38.10 (37.40, 38.60)	0.2
Systolic Blood pressure on day 0	120 (105, 135)	125 (105, 140)	0.085
On IV fluids on day 0	284 (44%)	84 (39%)	0.2
On vasopressor drugs on day 0	71 (11%)	16 (7.4%)	0.13
On corticosteroids on day 0, 1 or 2	67 (10%)	24 (11%)	0.7
Hospital acquired	265 (41%)	110 (51%)	0.01
Charlson score	3.0 (2.0, 5.0)	3.0 (2.0, 4.0)	0.3
Mental status			0.042
Grade I	17 (2.6%)	2 (0.9%)	
Grade II	26 (4.0%)	4 (1.8%)	
Grade III	41 (6.3%)	9 (4.1%)	
Grade IV	16 (2.5%)	1 (0.5%)	
None	551 (85%)	201 (93%)	
Time to appropriate therapy (hours)	3.0 (1.0, 26.0)	5.0 (0.0, 34.0)	0.14
Dead at 28 days	138 (21%)	30 (14%)	0.017

1 n (%); Median (IQR).

2 Pearson's Chi-squared test; Wilcoxon rank sum test; Fisher's exact test.

### Association between 28-day mortality and transplantation status

Regression models were built as described above for transplant status with output shown in Table 5 for both the whole cohort and the propensity score matched cohort. In both analyses, transplant status was associated with a significant reduction in 28-day mortality, with an odds ratio of 0.55 (95% CI 0.34 to 0.90) in the propensity score matched model and 0.47 (95% CI 0.28 to 0.76) in the whole cohort. However, in both models, corticosteroid use was strongly associated with increased mortality OR 5.24 (95% CI 2.79 to 9.84) in the PSM cohort, and OR 4.4 (95%CI 3.12 to 6.20) in the whole cohort.

**Table 5:** Output from the logistic regression models for both the whole cohort and the propensity score matched model.

	*Whole cohort*			*Propensity Score Matched*		
Characteristic	OR^1^	95% CI^1^	p-value	OR^1^	95% CI^1^	p-value
Transplant (univariable)	0.53	0.35, 0.78	0.002	0.59	0.38,0.93	0.023
Transplant (multivariable)	0.47	0.28, 0.77	0.004	0.55	0.34, 0.90	0.018
Corticosteroid usage	4.40	3.12, 6.20	<0.001	5.24	2.79, 9.84	<0.001
Age	1.03	1.02, 1.04	<0.001	1.02	1.00, 1.04	0.021
Charlson's Comorbidity Index (CCI)	1.20	1.15, 1.25	<0.001	1.23	1.07, 1.41	0.005
Recent chemotherapy	0.74	0.54, 1.00	0.054	0.75	0.41, 1.37	0.3
Infective Organism						
Coagulase negative staphylococci	–	–		–	–	
Anaerobes	1.12	0.34, 3.17	0.8			
Candida spp	1.97	0.92, 4.12	0.075	1.53	0.24, 9.92	0.7
Enterobacterales	1.01	0.70, 1.47	>0.9	1.35	0.47, 3.92	0.6
Enterococci	0.97	0.53, 1.76	>0.9	1.67	0.59, 4.73	0.3
Other	1.05	0.73, 1.51	0.8	0.75	0.21, 2.70	0.7
Polymicrobial	1.23	0.79, 1.92	0.4	1.39	0.45, 4.23	0.6
Pseudomonas spp	1.54	0.88, 2.67	0.13	1.13	0.29, 4.36	0.9
Staphylococcus aureus	1.62	1.04, 2.51	0.031	0.96	0.30, 3.05	>0.9
Streptococci	0.7	0.45, 1.08	0.11	0.59	0.15, 2.33	0.4
Hospital acquired infection	1.63	1.35, 1.97	<0.001	2.41	1.06, 5.44	0.038
Gender						
Female	–	–		–	–	
Male	1.01	0.85, 1.21	0.9	0.95	0.57, 1.56	0.8
Centre						
1 (reference)	–	–				
2	0.89	0.65, 1.22	0.5	1.37	0.63, 2.96	0.4
3	0.98	0.76, 1.25	0.8	0.80	0.39, 1.64	0.5
4	1.24	0.84, 1.82	0.3	*		
5	1.42	1.07, 1.89	0.016	1.60	0.77, 3.33	0.2
6	1.05	0.71, 1.52	0.8	1.33	0.54, 3.27	0.5
7	0.78	0.51, 1.18	0.2	0.45	0.08, 2.62	0.4
Focus of infection						
Contaminant (reference)						
Cardiovascular system	0.86	0.40, 1.77	0.7	0.49	0.01, 20.5	0.7
Gastrointestinal system	0.94	0.60, 1.46	0.8	0.62	0.16, 2.45	0.5
Line infection - central venous line	0.54	0.32, 0.88	0.016	0.46	0.13, 1.69	0.2
Lower respiratory tract	1.93	1.27, 2.94	0.002	3.10	1.02, 9.42	0.046
Site uncertain	1.73	1.22, 2.46	0.002	1.26	0.49, 3.27	0.6
Skin and soft tissue	0.61	0.33, 1.10	0.11	1.59	0.18, 13.8	0.7
Urinary tract infection	0.50	0.33, 0.77	0.002	0.30	0.09, 0.99	0.048
Other	0.47	0.26, 0.83	0.012	0.58	0.08, 4.05	0.6

*unreliable estimate, due to low numbers of cases at Centre 4.

1 OR = Odds Ratio, CI = Confidence Interval.

### Type of transplant and mortality

Given the small numbers, (e.g. 4 cases of heart transplant), it was impossible to reliably assess whether the type of transplantation affected mortality for most transplant types. However, as a sensitivity analysis we performed the regression model with type of transplant limiting to all transplants with more than 20 occurrences in the database. Table 6 shows the output of this model. Many results did not meet statistical significance which may be due to the low numbers, but for the two largest groups, HSCT (*n* = 99) and Kidney (*n* = 70), the effect size was almost exactly the same (Odds Ratio around 0.50 and 0.49 in the whole cohort, and 0.49 and 0.55 in the PSM cohort). As the effect of transplant might differ between SOT and HSCT, we re-ran the analyses comparing all SOT vs HSCT (Table S2). We found no evidence that the effect of transplant was different between SOT and HSCT patient. (OR 0.50; 0.27 to 0.87 for HSCT, OR 0.56; 0.32 to 0.92 in the whole cohort)

**Table 6:** Output of the individual transplant type model.

Characteristic	Whole cohort			Propensity Score Matched		
	OR	95%CI	p-value	OR	95%CI	p-value
No transplant	–	–	–	–	–	–
Bone marrow	0.50	0.27, 0.87	0.022	0.56	0.30, 1.05	0.070
Kidney	0.49	0.23, 0.94	0.048	0.55	0.26, 1.14	0.11
Liver	0.28	0.04, 0.94	0.082	0.31	0.07, 1.34	0.12
Other	1.25	0.45, 3.05	0.6	1.40	0.53, 3.66	0.5

1 OR = Odds Ratio, CI = Confidence Interval.

### Interaction with steroids

Given that steroids were associated with increased mortality, we performed two analyses to explore whether this effect was the same in transplant populations. In the first analysis (Table S3), we included an interaction between steroid usage and transplant status as a covariate. In both the whole and propensity score matched cohort, we did not find any significant interaction (*p* > 0.1 for both)

To aid clinical interpretation, we used a factorial design to compare those with no transplant and not on steroids (reference), to the three potential other groups (transplant and no steroid, transplant and steroid, steroid and non transplant). This is reported in Table S4, and shows the same findings – patients with organ transplant and no steroid use have remarkably reduced mortality (OR 0.28; 95% CI 0.26–0.30), while patients with organ transplant and steroids have similar risks (OR 3.07; 95% Ci 1.34–6.89) to those without organ transplant receiving steroids (OR 3.90; 95% CI 2.91–5.22). This suggests that both steroid use and transplant status are relevant for short term mortality.

### Mediation analysis

We performed an analysis to explore whether this reduced mortality was mediated by clinical variables on admission. In the whole cohort, the only clinical variable on admission that differed between the two groups was neutrophil count (lower, on average, in transplant patients).

We therefore performed a multivariate mediation analysis on neutrophil count pressure on the multiply imputed dataset, reported fully in the supplementary appendix. This model identified a significant causally mediated effect with an estimated proportion of effect explained of 15% (95% CI 5.9% to 59.7%, Supplementary Table 1).

However, given that the reduced neutrophil count was largely seen in patients with HSCT, we performed a sensitivity analysis stratifying on HSCT. In this analysis, a significant effect was only found in the HSCT groups (Supplementary Tables 2 and 3). This may represent either a true null association, or reflect a lack of statistical power due to the reduced sample size.

In summary, this suggests that at least part of the beneficial effect of organ transplantation on mortality in HSCT is mediated by a reduction in neutrophil count, although we did not identify this association in other organ transplants.

We planned to also mediate on time to appropriate therapy, however this did not significantly differ between those with organ transplant and those without.

## Discussion

This study shows that patients with organ transplant who are not receiving systemic corticosteroids have reduced mortality in bloodstream infection than patients without organ transplantation in bloodstream infection. The magnitude of the effect was large, and similar in both unadjusted and adjusted analyses.

Of all the clinical variables on presentation recorded, only neutrophil count differed between patients with organ transplant and those without, with lower neutrophils recorded in transplant patients. Formal mediation analysis suggests this neutropenia may mediate some of the beneficial effects of organ transplantation, although the association was limited to HSCT, and the interpretation of a single neutrophil count on admission is difficult without detailed data on the immunosuppressive regime and underlying haematological diagnosis.

Time to appropriate therapy was similar between groups, suggesting this benefit was not simply due to earlier treatment with appropriate antibiotics.

### Strengths and weaknesses

This study has many strengths. Firstly, the analysis was performed on prospectively collected, multi-centre, trial data, with relatively little missing data on both exposure and outcome. Infective pathogen, type of transplant, and clinical variables were taken directly from the clinical notes. Time to appropriate therapy was reliably recorded, as was the focus of infection. Secondly, our statistical approach aimed to reduce confounding by matching patients via a propensity score, and it is reassuring that both matched and unmatched models had broadly similar effects. Thirdly, the comparator population was all patients admitted with bloodstream infection at the same time, a pragmatic and clinically relevant comparison group. Finally, the relatively large number of patients included for a bloodstream infection trial (>4000), allow us confidence in our results.

Common to many observational studies, this study was limited by the lack of detailed information on type of transplant, type of immunosuppression, and chronicity of transplant, as well as detailed information on treatment. Most importantly, although we attempted to match patients based on age and comorbidities, likely at least some of the effect is driven by residual confounding by transplantation status. For example, small differences in rates of hospital acquired infection, and receipt of recent chemotherapy remained after matching. Also, rates of some transplants (e.g. lung) were low in this study, and we can be most confident in our findings in organ transplants where we have large numbers (e.g. kidney, liver, and HSCT). One key question – what is the mechanism of the association, and is the association causal, cannot be completely answered by this study, and is the focus of much of the discussion below. It is also important to recognise the impact of steroid usage on mortality. Receipt of corticosteroids had a large and robust association with an increased mortality, and we did not identify that this differed in patients with or without a transplant (p for interaction >0.1). Therefore, the data supporting reduced mortality in patients with transplant is should be interpreted with respect to steroid use, as those with steroid use may actually have a higher mortality with respect to those not on steroids and not receiving a transplant. However, this represented only a small (10%) number of patients in this cohort, but may represent larger amounts in other clinical settings. Finally, this trial recruited patients in 2013/14, and the management of patients with both solid organ transplant and HSCT has changed since then, with large improvements in mortality more generally in patients with HSCT.^8^

### Other literature

Previous work has examined this topic, with broadly similar findings. The most similar study in bloodstream infection, by Kalil et al.,^8^, ^9^, ^10^ included 123 patients with organ transplant and 246 matched controls. In Cox regression, the hazard for 28 day mortality was 0.22 (95% CI 0.9 −0.54), despite lower initial appropriate antimicrobial usage. In a large cohort of sepsis hospitalisations (903,816 hospitalisations, 39,618 with organ transplant) Donnelly et al., found inpatient mortality in organ transplant around half of that of controls in severe sepsis (5.5% vs 9.4%) and a third less in sepsis (8.7% vs 12.%). After adjusting for confounding, the relevant odds ratios were 0.83 (95% CI 0.79, 0.87) for severe sepsis, and 0.78 (95% CI 0.73 −0.84) for sepsis. In agreement, one small study in S. aureus bacteraemia showed a reduction in mortality with solid organ transplant, but two studies suggested higher mortality in lung transplant.^9^, ^10^, ^11^ Outside bloodstream infection, the data is much less clear, with studies in COVID-19^12^ and pneumonia suggesting both higher and lower mortality in transplant recipients.^12^

In summary, our work, in conjunction with the other literature, supports lower short-term mortality in patients with a bloodstream infection specifically, with all three large studies identifying a decrease in short term mortality in patients with organ transplantation. There are four broad potential reasons.

Firstly, residual confounding is likely to play a part. Although in this study (and others), patients were matched by age and comorbidity, it is probable that patients with organ transplant are somewhat “fitter” than their matched cohort, given that a certain level of medical fitness is generally required to have an organ transplant, and we could not match for all comorbidities. Although this effect is likely present, the lack of change in estimate in our study from the whole cohort to the adjusted model suggests this effect may be marginal, although we cannot rule out residual confounding.

Secondly, patients with organ transplant may present differently, with a possibility of earlier presentation leading to earlier subsequent antimicrobial therapy and better outcomes.^13^ Although this is possible, we have previously shown that “time to positivity”, a proxy for bacterial load, is no different in transplant patients than non-transplant patients in this cohort, and time to appropriate therapy in this study was the same across both cohorts.^14^ Also, in this cohort, infections with Pseudomonas spp (associated with worse outcomes) were higher in the transplant cohort, while coagulase negative staphylococci (often felt to be marginally pathogenic) were higher in the non-transplant cohort. Regardless, given that early therapy improves outcomes in bloodstream infection, even small changes in patient behaviour (e.g. earlier presentation) could account for some of the mortality difference

Secondly, patients with organ transplant are treated differently to patients without organ transplant, generally receiving specialist care. The impact of this care can be hard to quantify, but it is likely (although not proven) that specialist units generally have better outcomes than generalist units, and at least some of the decreased mortality may be due to specialist care, outside antimicrobial therapy. In particular, usage of appropriate antimicrobials is likely to be greater, as the microbiology of BSI in patients with organ transplant is different, with specialist centres perhaps more likely to prescribe both empiric and longer term therapy that is more appropriate. Secondly, it is well established that supportive care (e.g. VTE prophylaxis, fluid management, regular re-assessment of patients), is a critical determinant of survival from infection; and it is highly plausible that these practices differ in specialist centres. There is evidence that consultation with infectious disease services improves some outcomes in organ transplantation, and there are now multiple papers suggesting decreased mortality for numerous infectious diseases associated with specialist care, although these studies themselves are observational and subject to some bias.^15^, ^16^, ^17^

Finally, it is likely that patients who have an organ transplant manage infection differently to those who do not, due to the presence of immunosuppression. The results of the mediation analysis hint at this, with lower neutrophil counts being causally associated with improved outcomes, although this should be seen as an exploratory analysis only given the association was only identified in those with HSCT. This analysis was performed as neutrophilia is known to be associated with worse outcomes in bloodstream infection, and it may be by that by artificially reducing neutrophil count some of the adverse effects immunological sequalae associated with bloodstream infection can be mitigated.

However, the association with neutrophil count in HSCT is complex, as infections at different periods of the engraftment are likely to have dramatically differing short and long term outcomes, and the apparent association with neutrophil count may well not be causal

The benefits of immunosuppression in some types of infection are well established, with the recent RECOVERY trial showing the benefits of steroids in patients with COVID-19 requiring hospitalization, with supporting evidence also in diverse conditions such as PCP pneumonia^18^ and TB meningitis,^19^ while the role of steroids for sepsis and community acquired pneumonia remains controversial.^20^, ^21^, ^22^

This work adds to the literature suggesting that immune status has a complex relationship with infection, and that immunosuppression may not always be harmful in patients with severe infection.

It is also worth noting that in this study, receipt of steroids on admission or day 1 or 2 was associated with an increased mortality, and that this was also present in those who had organ transplant suggesting that the interaction between steroid use and beneficial outcomes in bloodstream infection is complex and multi-faceted. However, without knowing the indication for the steroids (e.g. rejection, Graft-versus-host disease), interpretation of this data is difficult, but this reduced mortality is likely only applicable to those not receiving systemic corticosteroids. In some settings, (e.g. peri‑engraftment) steroid use is much more common than in this cohort, and caution should be used extrapolating outside this setting.

The importance of determining which of these reasons is most relevant remains important. If much of the benefit can be explained by earlier presentation and higher quality of care for patients who have had organ transplant this suggests that we can improve outcomes in other patients with bloodstream infection. However, if the cause is due to a degree of immune tolerance for foreign pathogens, this suggest that therapeutic immunosuppression may represent a future therapeutic avenue for patients with bloodstream infection.

## Conclusion

Patients with organ transplantation who are not on systemic corticosteroids have a markedly reduced short-term mortality in bloodstream infection. Understanding the mechanisms and whether this represents a causal association are key future questions for researchers.

## Funding

FH's time was funded by the GW4 Wellcome Doctoral Fellowship programme. PG was supported by the Ser Cymru programme funded by the Welsh Government and the EU ERDF.

The 10.13039/501100000272National Institute for Health Research (NIHR) Programme Grants for Applied Research funded the RAPIDO trial (RP-PG-0707–10,043). The views and opinions expressed are those of the authors and do not necessarily reflect those of the NIHR HTA programme, the NIHR, the UK NHS or the Department of Health.

## Declaration of Competing Interest

The authors declare that they have no known competing financial interests or personal relationships that could have appeared to influence the work reported in this paper.

## Appendix Supplementary materials

Image, application 1
