# Supplementary material for: Patients with transplantation have reduced mortality in bacteraemia: Analysis of data from a randomised trial
Source: J Infect. 2022 Jul;85(1):17–23. doi: 10.1016/j.jinf.2022.05.014 (PMC9646478; doi:10.1016/j.jinf.2022.05.014)
Supplement: Supplementary file 1 [file mmc1.docx]

Table S1: Microbiological matching on the imputed cohort:

|  | No transplant  n = 651 | Transplant  n = 217 |
| --- | --- | --- |
| Organism: |  | |
| CoNS | 142 (22%) | 45 (21%) |
| Anaerobes | 0 (0%) | 0 (0%) |
| Candida spp | 8 (1.2%) | 3 (1.4%) |
| Enterobacterales | 211 (32%) | 74 (34%) |
| Enterococci | 42 (6.5%) | 16 (7.4%) |
| Other | 57 (8.8%) | 16 (7.4%) |
| Polymicrobial | 51 (7.8%) | 18 (8.3%) |
| Pseudomonas spp | 46 (7.1%) | 14 (6.5%) |
| Staphylococcus aureus | 45 (6.9%) | 14 (6.5%) |
| Streptococci | 49 (7.5%) | 17 (7.8%) |
| Focus |  | |
| Bone and joint | 1 (0.2%) | 1 (0.5%) |
| Cardiovascular system | 7 (1.1%) | 3 (1.4%) |
| Central nervous system | 7 (1.1%) | 2 (0.9%) |
| Eye, ear, nose, throat or mouth | 5 (0.8%) | 1 (0.5%) |
| Gastrointestinal system | 56 (8.6%) | 20 (9.2%) |
| Line infection - central venous line | 120 (18%) | 41 (19%) |
| Line infection - peripheral venous line | 4 (0.6%) | 1 (0.5%) |
| Lower respiratory tract | 51 (7.8%) | 15 (6.9%) |
| N/A - contaminant | 86 (13%) | 30 (14%) |
| Reproductive tract | 0 (0%) | 0 (0%) |
| Site uncertain | 183 (28%) | 61 (28%) |
| Skin and soft tissue | 10 (1.5%) | 3 (1.4%) |
| Surgical site infection | 4 (0.6%) | 2 (0.9%) |
| Systemic Infection | 0 (0%) | 0 (0%) |
| Urinary tract infection | 117 (18%) | 37 (17%) |

Table S2: Analysis of SOT vs HSCT

|  | Whole cohort | | | Propensity Score Matched | | |
| --- | --- | --- | --- | --- | --- | --- |
|  | OR^1^ | 95% CI^1^ | p-value | OR | 95% CI | p-value |
| No transplant | — | — |  | — | — |  |
| HSCT | 0.50 | 0.27, 0.87 | 0.022 | 0.56 | 0.30, 1.04 | 0.067 |
| SOT | 0.56 | 0.32, 0.92 | 0.029 | 0.62 | 0.35, 1.09 | 0.10 |
| 1 OR = Odds Ratio, CI = Confidence Interval | | | | | | |

Mediation analysis:

Table S3: Interaction between steroids and transplant status

| Characteristic | Whole cohort | | | Propensity Score Matched | | |
| --- | --- | --- | --- | --- | --- | --- |
|  | OR^1^ | 95% CI^1^ | p-value | OR^1^ | 95% CI^1^ | p-value |
| Corticosteroid use | 3.90 | 2.91, 5.22 | <0.001 | 5.63 | 3.19, 9.93 | <0.001 |
| Transplant recipient | 0.40 | 0.24, 0.62 | <0.001 | 0.50 | 0.30, 0.85 | 0.010 |
| Corticosteroid use * Transplant recipient | 1.99 | 0.74, 5.32 | 0.2 | 1.38 | 0.46, 4.10 | 0.6 |
| 1 OR = Odds Ratio, CI = Confidence Interval | | | | | | |

Table S4: Factorial analysis of steroids and transplant

| Characteristic | Whole cohort | | | Propensity Score Matched | | |
| --- | --- | --- | --- | --- | --- | --- |
|  | OR^1^ | 95% CI^1^ | p-value | OR^1^ | 95% CI^1^ | p-value |
| no transplant/steroids | — | — |  | — | — |  |
| Transplant alone | 0.40 | 0.24, 0.62 | <0.001 | 0.50 | 0.30, 0.85 | 0.010 |
| Steroids alone | 3.90 | 2.91, 5.22 | <0.001 | 5.63 | 3.19, 9.93 | <0.001 |
| Steroids and transplant | 3.07 | 1.34, 6.89 | 0.006 | 3.88 | 1.69, 8.95 | 0.001 |
| 1 OR = Odds Ratio, CI = Confidence Interval | | | | | | |

Mediation analysis was performed using the *mediate* package in R. A multivariable mediation analysis was performed on the imputed dataset, with the exact code shown here for the neutrophil analysis, and full outputs below.

mediation_function_matched_neutrophils<- function(x) {

fit_dv <- glm(death ~ bug_group + age + hosacq + charl + transplant + cortico + centreid + neutrophil,

data = x ,

family = "binomial")

fit_mediate <- lm(neutrophil ~ transplant, data = x)

mediation_results <- mediate(fit_mediate, fit_dv, treat = "transplant", mediator = "neutrophil")

results <- summary(mediation_results)

return(results)

}

Table S1: Mediating effect of neutrophils between transplantation and death (whole cohort)

| Term | Estimate | Lower 95% CI | Upper 95% CI | p.value |
| --- | --- | --- | --- | --- |
| Average causally mediated effect (no transplant) | −0.014 | −0.023 | −0.006 | <0.001 |
| Average causally mediated effect (transplant) | −0.011 | −0.020 | −0.004 | <0.001 |
| Average direct effect  (no transplant) | −0.065 | −0.113 | −0.009 | 0.02 |
| Average direct effect  (no transplant) | −0.062 | −0.1078 | −0.008 | 0.02 |
| Average proportion mediated | 0.16 | 0.06 | 0.56 | 0.006 |

Table S2: Mediating effect of neutrophils between transplantation and death (excluding bone marrow transplants)

| Term | Estimate | Lower 95% CI | Upper 95% CI | p.value |
| --- | --- | --- | --- | --- |
| Average causally mediated effect (no transplant) | −0.003 | −0.010 | 0.004 | 0.38 |
| Average causally mediated effect (transplant) | −0.002 | −0.008 | 0.003 | 0.38 |
| Average direct effect  (no transplant) | −0.073 | −0.134 | −0.004 | 0.038 |
| Average direct effect  (no transplant) | −0.072 | −0.132 | −0.004 | 0.038 |
| Average proportion mediated | 0.03 | -0.07 | 0.26 | 0.39 |

Table S3: Mediating effect of neutrophils between transplantation and death (only bone marrow transplants)

| Term | Estimate | Lower 95% CI | Upper 95% CI | p.value |
| --- | --- | --- | --- | --- |
| Average causally mediated effect (no transplant) | −0.026 | −0.041 | −0.013 | <0.001 |
| Average causally mediated effect (transplant) | −0.022 | −0.038 | −0.001 | <0.001 |
| Average direct effect  (no transplant) | −0.048 | −0.119 | 0.0434 | 0.25 |
| Average direct effect  (no transplant) | −0.044 | −0.108 | 0.0398 | 0.25 |
| Average proportion mediated | 0.299 | -1.586 | 2.32 | 0.1 |

Table S3: Mediating effect of systolic blood pressure between transplantation and death (only bone marrow transplants)
